# Supplementary figures and images for: Even good bots fight: The case of Wikipedia
Source: PLoS One. 2017 Feb 23;12(2):e0171774. doi: 10.1371/journal.pone.0171774 (PMC5322977; doi:10.1371/journal.pone.0171774)

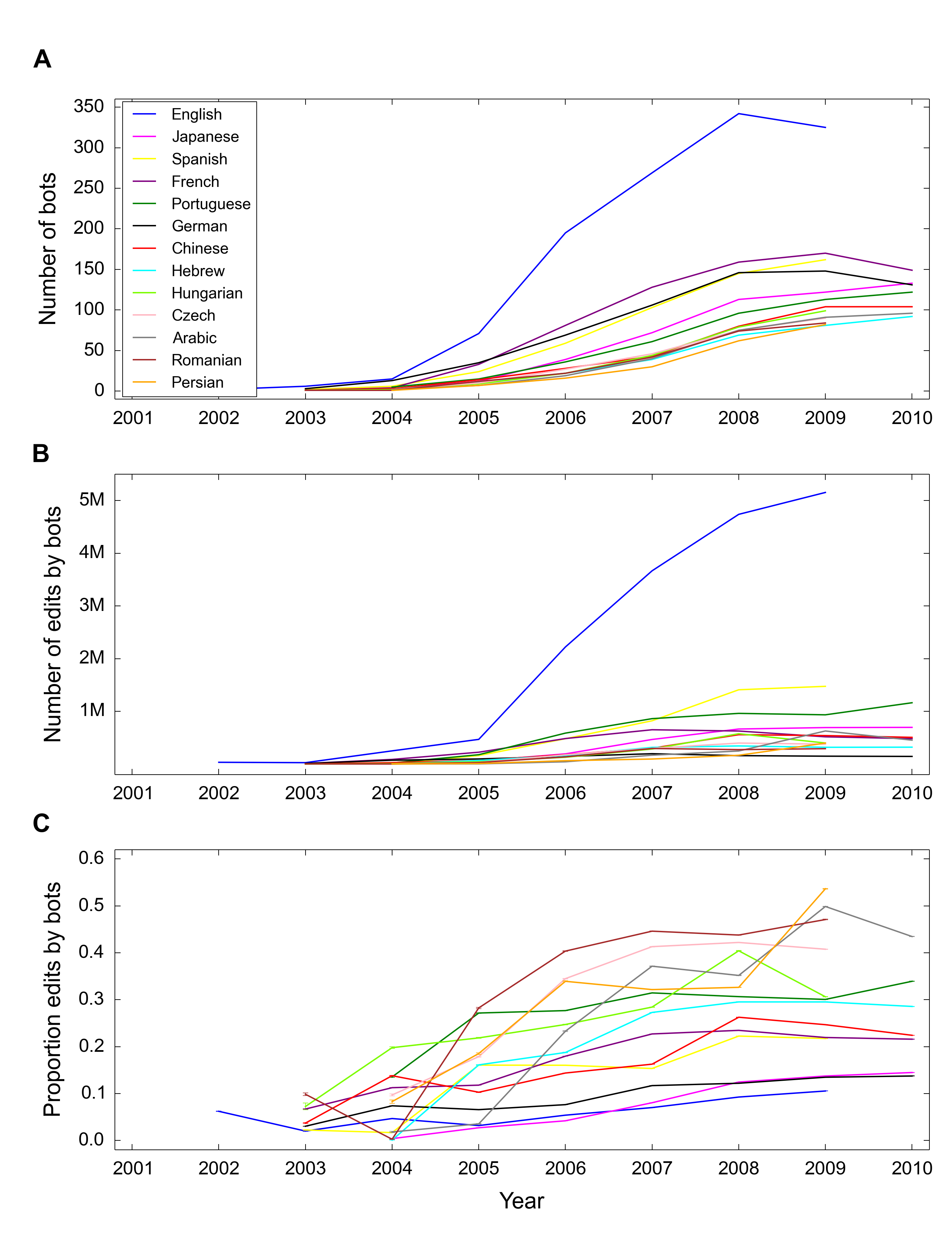

Supplement: S1 Fig — Between 2003 and 2008 the number of bots and their activity have been increasing. This trend, however, appears to have subsided after 2008, suggesting that the system may have stabilized. (TIFF) [file pone.0171774.s001.tiff]

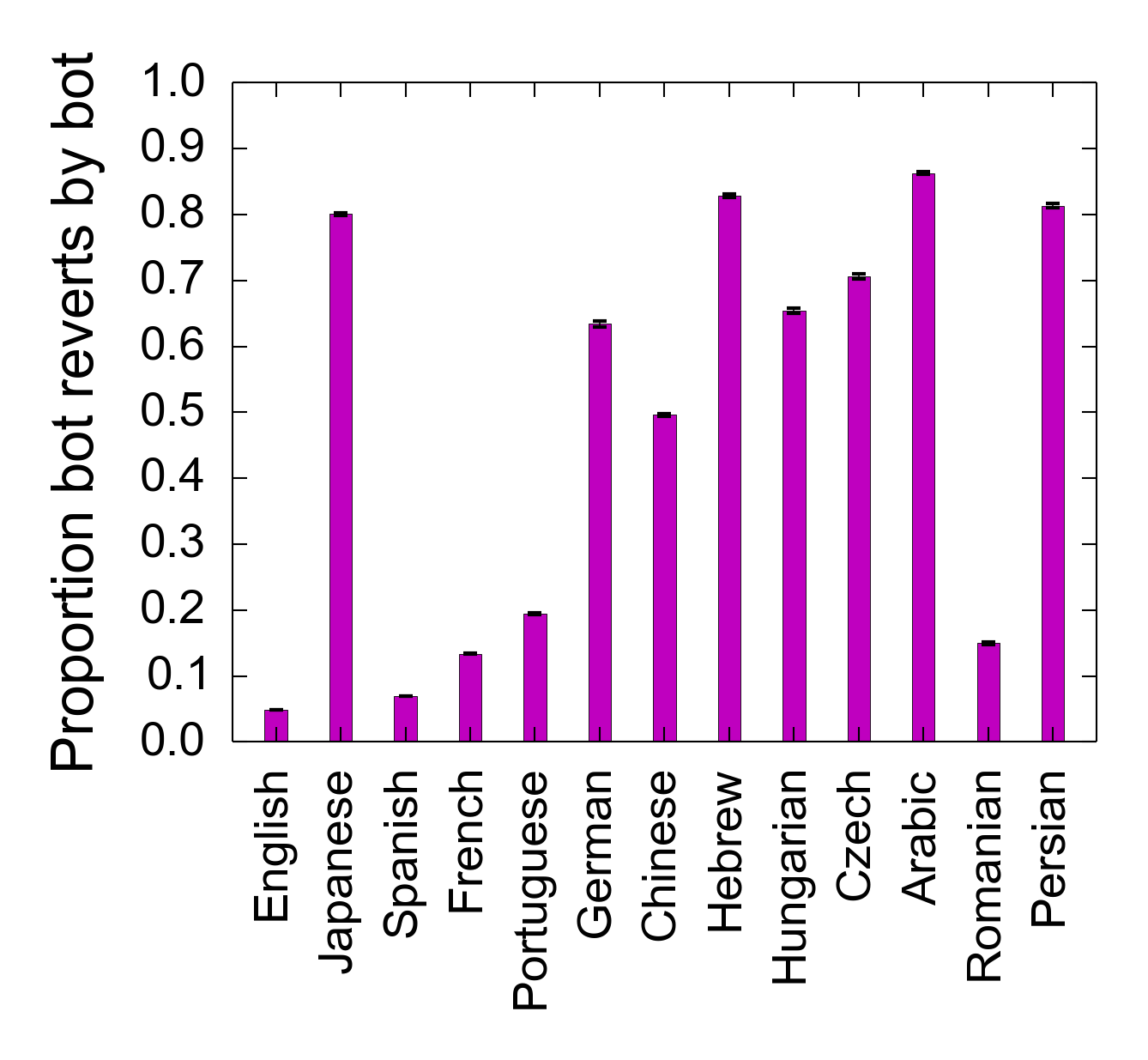

Supplement: S2 Fig — English and the Romance languages in our data present exceptions, with less than 20% of bot reverts are done by other bots. (TIFF) [file pone.0171774.s002.tiff]

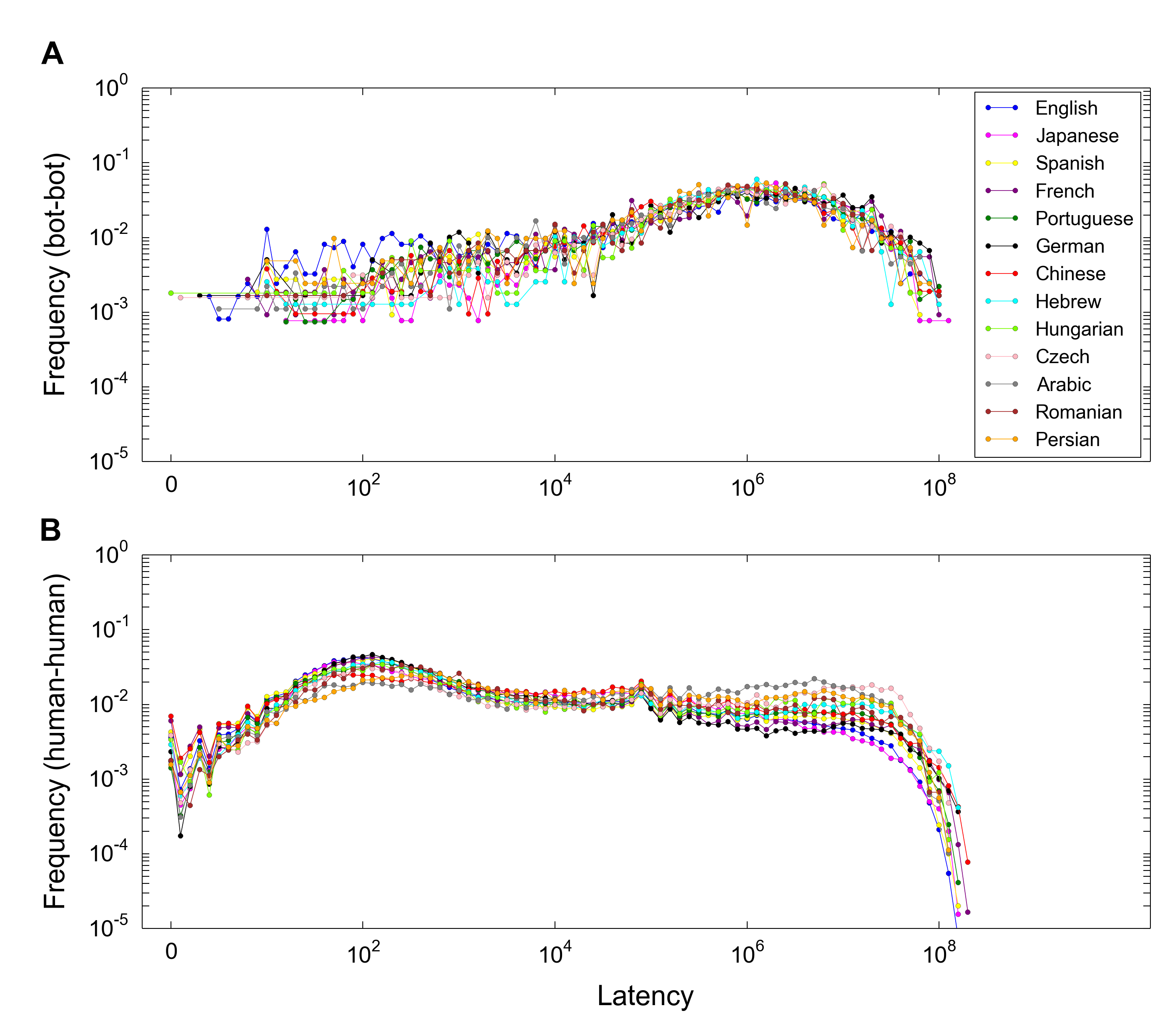

Supplement: S3 Fig — The figures show the distribution of interactions for a particular latency, where we define latency as the mean log time in seconds between successive reverts. (A) Bot-bot interactions have a characteristic latency of 1 month, as indicated by the peak in the figure. (B) Human-human interactions occur with a latency of 2 minutes, 24 hours, or 1 year. (TIFF) [file pone.0171774.s003.tiff]

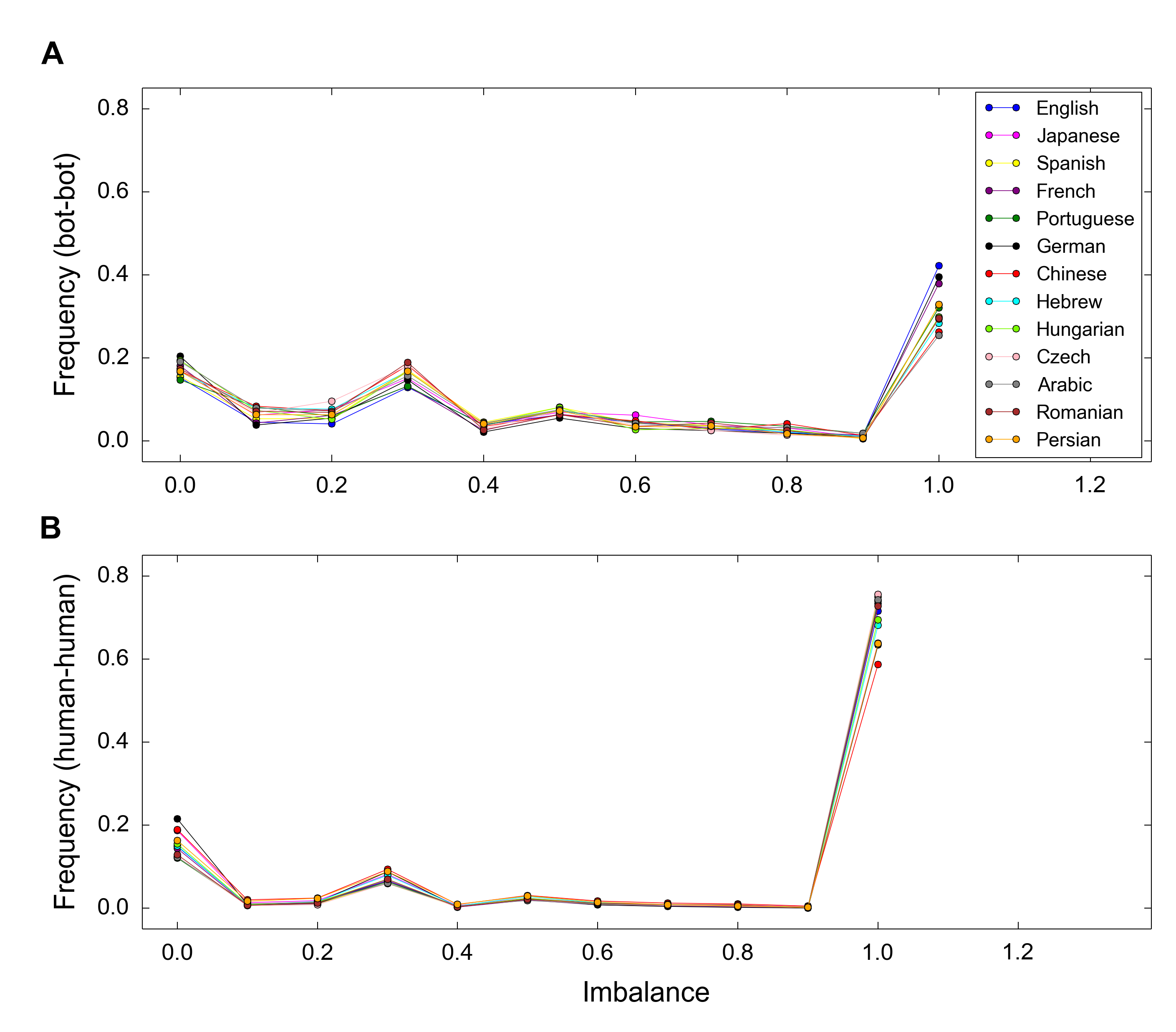

Supplement: S4 Fig — We define imbalance as the final proportion of reverts between i and j that were not reciprocated. (A) A significant proportion of bot-bot interactions have low imbalance. (B) The majority of human-human interactions are perfectly unbalanced. (TIFF) [file pone.0171774.s004.tiff]

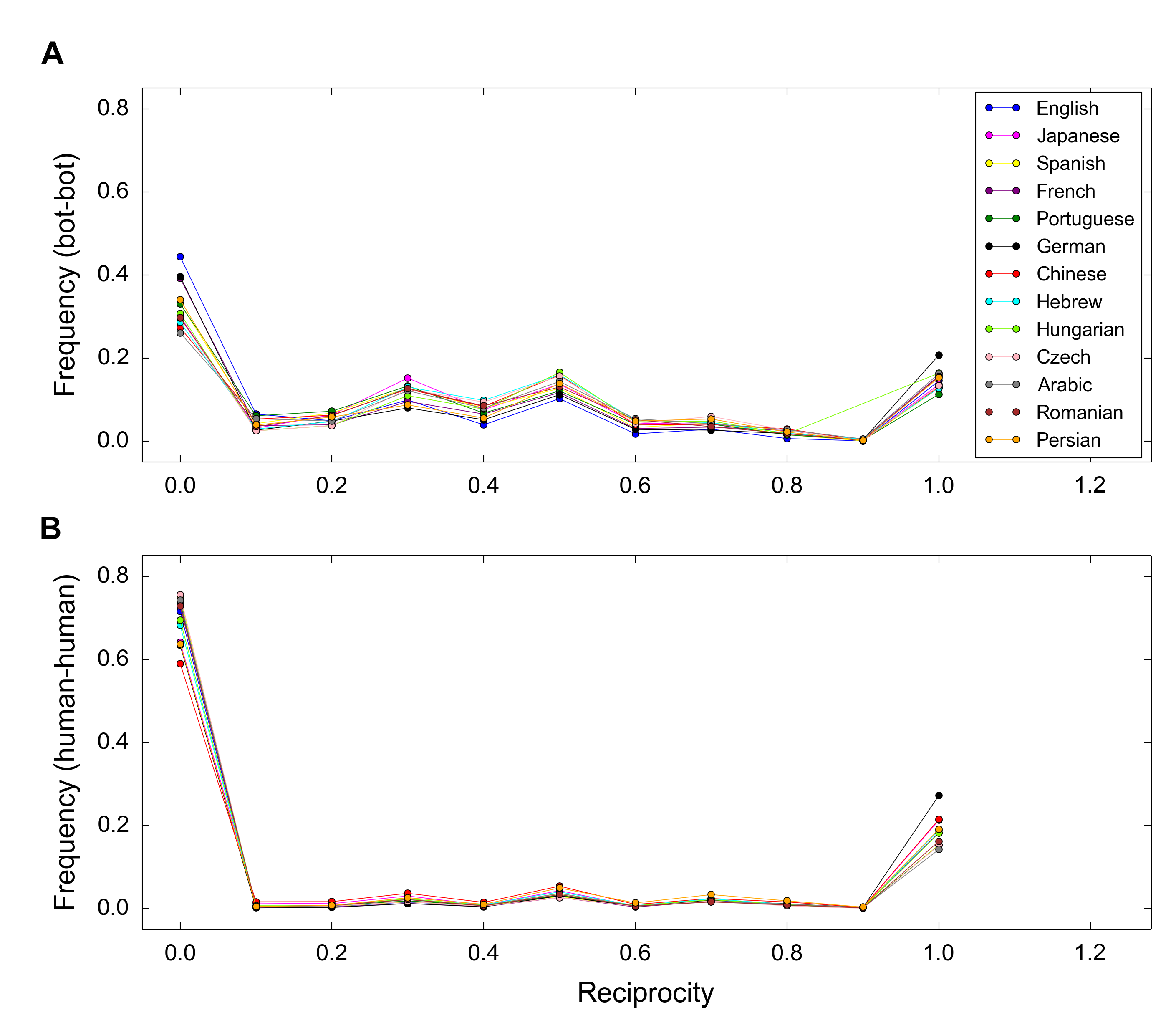

Supplement: S5 Fig — We measure reciprocity as the proportion of observed turning points out of all possible. (A) A significant proportion of bot-bot interactions have intermediate or high values of reciprocity. (B) The majority of human-human interactions are not reciprocated. (TIFF) [file pone.0171774.s005.tiff]

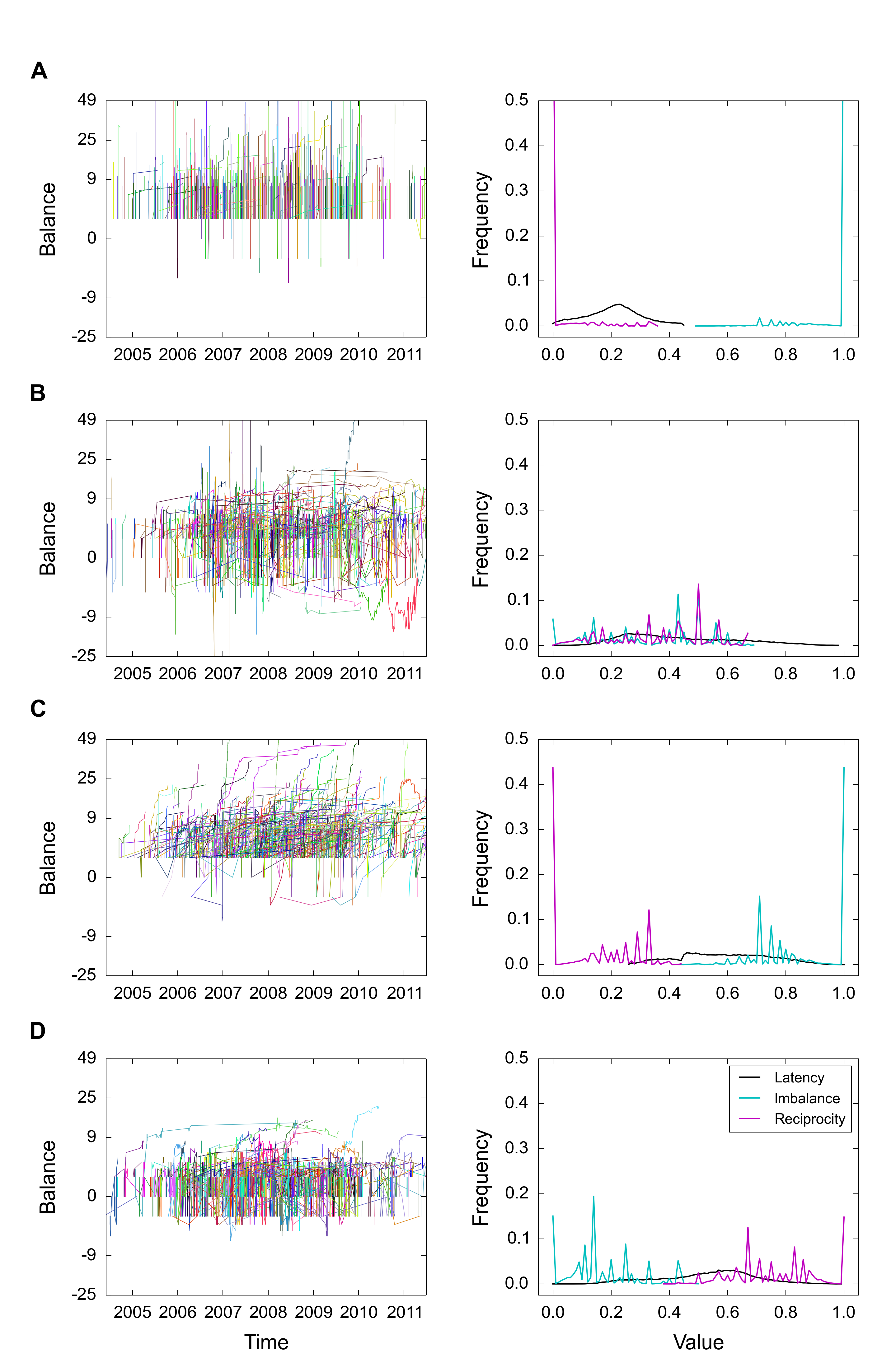

Supplement: S6 Fig — The left panels show a sample of the trajectories, including bot-bot and human-human interactions and trajectories from all languages. The right panels show the distribution of latency, imbalance, and reciprocity for each type of trajectory. The three properties measure the average steepness, the y-value of the last point, and the jaggedness of the trajectory, respectively. (A) Fast unbalanced trajectories have low reciprocity and latency and high imbalance. (B) Somewhat balanced trajectories have intermediate imbalance and reciprocity. (C) Slow unbalanced trajectories have low reciprocity and high latency and imbalance. (D) Well balanced trajectories have low imbalance and high reciprocity. (TIFF) [file pone.0171774.s006.tiff]

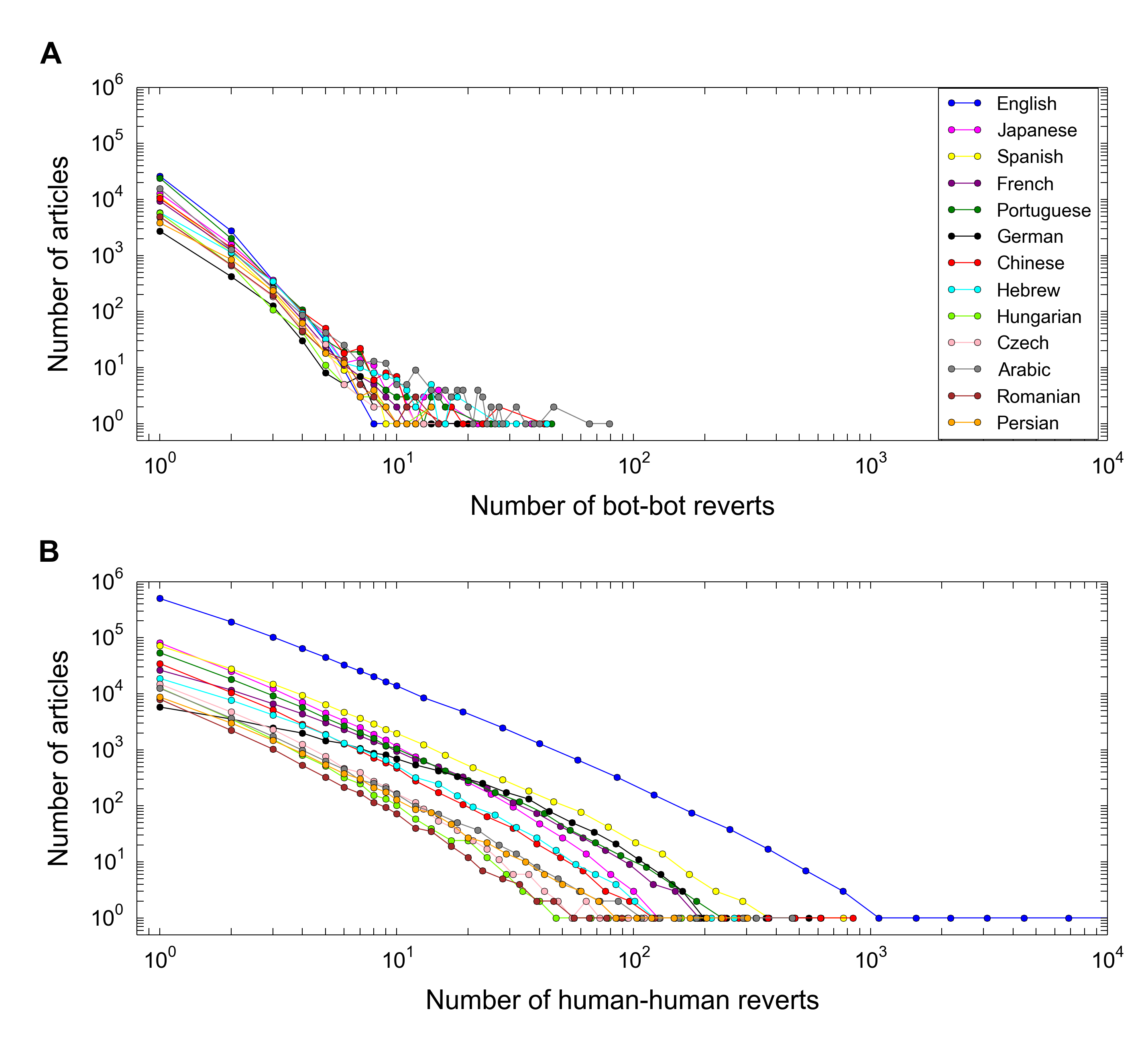

Supplement: S7 Fig — (A) Few articles include more than 10 bot-bot reverts. The most contested articles tend to be about foreign countries and personalities. Further, the same articles also re-appear in different languages. (B) There are many articles that are highly contested by humans. The most contested articles tend to concern local personalities and entities. It is rare that a highly contested article in one language will be also highly contested in another language. (TIFF) [file pone.0171774.s007.tiff]

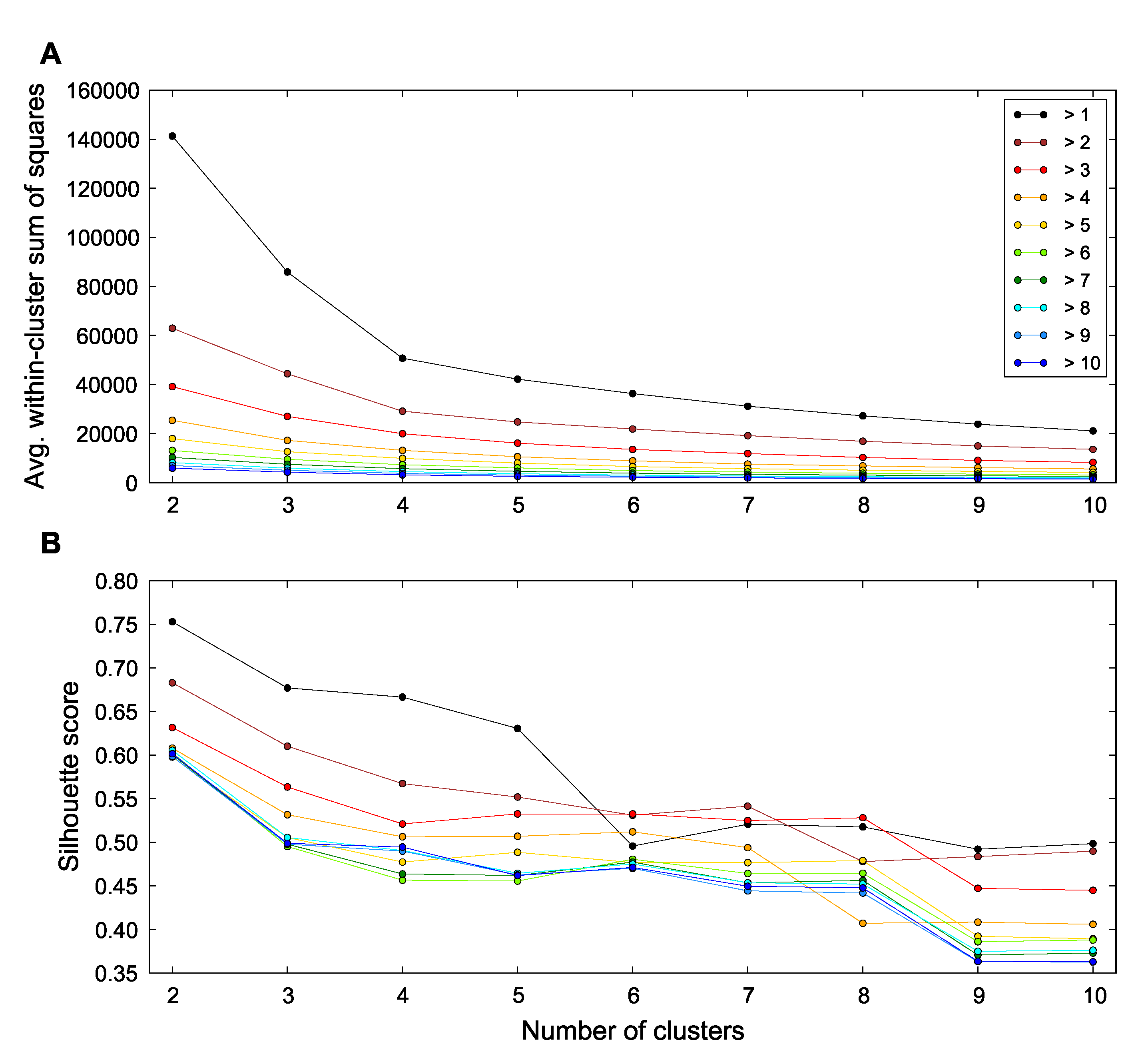

Supplement: S8 Fig — (A) The elbow method requires the smallest k that most significantly reduces the sum of squared errors for the clustering. Here, the method suggests that four clusters give the best clustering of the data. (B) The silhouette method requires the k that maximizes the separation distance between clusters, i.e. the largest silhouette score. Here, the method suggests that the clustering performs worse as the number of clusters increases. (TIFF) [file pone.0171774.s008.tiff]
