# Supplementary material for: Even good bots fight: The case of Wikipedia
Source: PLoS One. 2017 Feb 23;12(2):e0171774. doi: 10.1371/journal.pone.0171774 (PMC5322977; doi:10.1371/journal.pone.0171774)
Supplement: S1 Table — Bots revert each other to a great extent. They also reciprocate each other’s reverts to a considerable extent. Their interactions are not as clustered as for human editors. Still, both for bots and humans, more senior editors tend to revert less senior editors, as measured by node assortativity by number of edits completed. (PDF) [file pone.0171774.s009.pdf]

S1 Table. Descriptive statistics for the bot-bot layer and the human-human layer in the multi-layer networks of reverts.

	Number of nodes	Avg. number reverts per node	Reverts / edits	Prop. dyads with at least one revert reciprocated	Assortativity by number of edits	Avg. clustering	Avg. clustering / avg. clustering in random network
**Bot-bot**							
English	319	104.6	0.002	0.46	-0.02	0.43	19
Japanese	182	100.4	0.006	0.57	-0.13	0.58	9
Spanish	204	71.7	0.003	0.53	-0.06	0.57	12
French	225	59.3	0.004	0.47	-0.1	0.5	12
Portuguese	164	185	0.006	0.57	-0.12	0.64	8
German	178	24.1	0.004	0.43	-0.1	0.4	10
Chinese	151	103	0.006	0.59	-0.16	0.62	9
Hebrew	124	83.9	0.006	0.59	-0.11	0.59	7
Hungarian	116	66.8	0.004	0.54	-0.13	0.6	8
Czech	122	59	0.005	0.57	-0.18	0.56	8
Arabic	132	161.7	0.011	0.6	-0.05	0.6	7
Romanian	104	70.8	0.005	0.55	-0.11	0.6	7
Persian	106	63.8	0.005	0.5	-0.05	0.53	8
**Human-human**							
English	4127880	3.1	0.079	0.09	-0.05	0.04	72370
Japanese	193203	2.6	0.026	0.16	-0.05	0.02	2971
Spanish	508815	2.3	0.070	0.09	-0.11	0.1	33433
French	181395	2.6	0.045	0.09	-0.02	0.04	4011
Portuguese	262293	2.3	0.066	0.09	-0.14	0.12	19762
German	206734	2.2	0.069	0.11	-0.1	0.03	3703
Chinese	66470	3.2	0.028	0.18	-0.14	0.08	3377
Hebrew	70816	2.9	0.047	0.13	-0.13	0.2	7458
Hungarian	21036	2.4	0.016	0.11	-0.13	0.1	1265
Czech	23792	3.1	0.035	0.1	-0.18	0.19	2262
Arabic	39083	2.2	0.044	0.08	-0.17	0.11	2947
Romanian	16625	2.1	0.027	0.1	-0.2	0.11	1371
Persian	18657	3.6	0.056	0.16	-0.13	0.21	1972

Bots revert each other to a great extent. They also reciprocate each other's reverts to a considerable extent. Their interactions are not as clustered as for human editors. Still, both for bots and humans, more senior editors tend to revert less senior editors, as measured by node assortativity by number of edits completed.
